# A Case of Purpura Urticans

**Published:** 1860-09

**Authors:** Wm. H. Douhhty


*A Case of Purpura Urticans.* By WM. H. DOUHHTY, M. D.

Purpura, in any of its forms, its simplest as well as its most aggravated and alarming, is an extremely interesting disease—the more so, perhaps, because of its rarity and its insidiousness. Persons, apparently in the best of health, are sometimes made its unaccountable subjects, and when so occurring, as in the two cases reported in the Medical and Surgical Reporter of 28th July, it certainly appears a great anomaly. To those who are accustomed to view it as a blood disease, this is the more striking, since the altered condition of that menstruum which it is supposed to indicate, is such as to warrant the presumption of a slow, progressive agent or cause, whose destructive results have been attained by long continued influence.

The following is an imperfect history of a case of Purpura Urticans, which came under our supervision last fall, in which its sudden appearance was well marked.

The subject was a female child, about five years of age, of good general health, of bilious lymphatic temperament, and descended from parents of good health and in good circumstances. About September 1st, she was observed to limp in running, and to manifest some fatigue under her usual exercise. An examination was instituted by the mother, who discovered several whitish, rounded elevations, with hardened and reddened basis, and somewhat sore to the touch, on the inner side of the knee. No particular importance was attached to them, and simple frictions with spirits of camphor used for the relief of the soreness. Instead, however, of disappearing, these elevations degenerated into pure hemorrhagic discolorations and were soon followed by numerous others of different sizes, but confined entirely to the lower extremities.

At this time we were called, and found the following state of things: The general health of the child somewhat impaired, face pale and disposed to swell; eyes heavy, with dark shades under them; complexion muddy and sallow; tongue furred white, from tip to base; irregular appetite with tendency to constipation; slight febrile excitement, with wandering pains of some severity through the back and extremities. An examination of the lower extremities revealed a number of hemorrhagic spots varying in appearance, and extending from the ankle to the groin. Some were disappearing with the yellow discoloration of ecchymosis; there were others of a deeply livid venous hue, of large size; and others, again, possessing the character of those first described, were just forming. The latter were always succeeded by the former, and when pressed upon at any stage, evinced but little tenderness. Some of the elevations resembled closely the wheals and elevations of nettle-rash, yet were deficient in the excessive pruritus common to that disease, and, moreover, instead of vanishing rapidly, left permanent discolorations. This was the course of many of these purpural spots, though some were primitive effusions of blood under and through the integuments. The largest of the latter that we saw, was about two and a half inches in diameter, and was formed in the course of two or three hours. But few were ever seen upon the body, and only one on the inner side of the arm just above the elbow. These spots continued to form for five or six weeks in irregular succession, and during that time the patient remained in a more or less feeble and delicate state. In the afternoon the feet would be swollen sufficiently to prevent her from walking.

The treatment of the case was as follows: Hyd. c. Creta 10 grs. at night, to be followed the next morning by Epsom salts and magnesia, in suitable doses, so as to free the primae viae, correct acidity and alter the secretions of the alimentary canal. Throughout the entire period, the warm hip bath was used twice daily, to reduce feverishness, induce a free action of the skin, and for the relief of the wandering pains. Besides this, ferri muriatis tinct. gtts. vj. was administered in a small quantity of blackberry wine, three times daily. At the end of two weeks, little or no benefit had resulted from this course; the spots were still occurring and disappearing as before. Iodine and Sarsaparilla, in the proportion of three drops of Tinct. Iodinii and ℥ i Syr. Sarsapar. Comp. were then substituted for the other medicinal or constitutional treatment, and continued three weeks or more; at the end of which time every trace of the disease was removed, and the system of the child completely renovated.

When first called to this case, we were perplexed in a measure to know what to call it, as it resembled somewhat both Urticaria and Purpura. But upon referring to " Wilson on diseases of the skin," we were at once enabled to give it the above classification, the essential disease being Purpura and the urticarious appearances a mere modification. To the Sarsaparilla in the above treatment, we believe great efficiency must be accorded. We do not believe that the Iodine alone would have displayed such signal effects. We have used both agents alone repeatedly and have been greatly pleased with their effects in appropriate cases. But when combined, these effects are much enhanced.—In the combination we prefer the Tr. Iodine to any other preparation, even the Iodide of Potassium. The Radix Sarsaparillæ used in the preparation of the compound syrup was that indigenous to the Southern States, the Georgia Sasaparilla, as we have derisively heard it called. With us it has entirely superceded all other kinds, and from a somewhat extensive use of it, we can bear testimony to its superior efficacy in all cases requiring such an alterative and eliminating agent. We usually prepare the syrup ourselves, and employ the Stillingia in combination with it. The present disrepute of the preparations of Sarsaparilla is to a great extent attributable to the inert articles kept and prepared by druggists. My experience warrants me in asserting, that if physicians would prepare such articles themselves, they would be far oftener pleased than disappointed with their effects.

Finally in the cases reported by Dr. Calhoun, an incidental allusion was made to the cause of Purpura. "*Vegetables formed no part of the diet of either of the patients.*" The diet in the case reported by us was not quite so exclusive, though not far removed; meats, pastry and fruit, with very few vegetables, were the preferred diet of the child. After treatment was commenced, rice, hominy and such like, were chiefly advised, not, however, under any apprehension that an absence of vegetable food had anything to do with the causation of the disease.—*Medical & Surgical Reporter.*

